# Mineralocorticoid Receptor Signaling in the Inflammatory Skeletal Muscle Microenvironments of Muscular Dystrophy and Acute Injury

**DOI:** 10.3389/fphar.2022.942660

**Published:** 2022-06-28

**Authors:** Zachary M. Howard, Chetan K. Gomatam, Arden B. Piepho, Jill A. Rafael-Fortney

**Affiliations:** Department of Physiology and Cell Biology, College of Medicine, The Ohio State University, Columbus, OH, United States

**Keywords:** aldosterone, muscular dystrophy, inflammation, myeloid cells, skeletal muscle, mineralocorticoid receptor, spironolactone, eplerenone

## Abstract

Duchenne muscular dystrophy (DMD) is a striated muscle degenerative disease due to loss of functional dystrophin protein. Loss of dystrophin results in susceptibility of muscle membranes to damage, leading to muscle degeneration and continuous inflammation and fibrosis that further exacerbate pathology. Long-term glucocorticoid receptor (GR) agonist treatment, the current standard-of-care for DMD, modestly improves prognosis but has serious side effects. The mineralocorticoid receptor (MR), a ligand-activated transcription factor present in many cell types, has been implicated as a therapeutic target for DMD. MR antagonists (MRAs) have fewer side effects than GR agonists and are used clinically for heart failure. MRA efficacy has recently been demonstrated for DMD cardiomyopathy and in preclinical studies, MRAs also alleviate dystrophic skeletal muscle pathology. MRAs lead to improvements in muscle force and membrane stability and reductions in degeneration, inflammation, and fibrosis in dystrophic muscles. Myofiber-specific MR knockout leads to most of these improvements, supporting an MR-dependent mechanism of action, but MRAs additionally stabilize myofiber membranes in an MR-independent manner. Immune cell MR signaling in dystrophic and acutely injured normal muscle contributes to wound healing, and myeloid-specific MR knockout is detrimental. More research is needed to fully elucidate MR signaling in striated muscle microenvironments. Direct comparisons of genomic and non-genomic effects of glucocorticoids and MRAs on skeletal muscles and heart will contribute to optimal temporal use of these drugs, since they compete for binding conserved receptors. Despite the advent of genetic medicines, therapies targeting inflammation and fibrosis will be necessary to achieve optimal patient outcomes.

## Introduction: Duchenne Muscular Dystrophy and Chronic Inflammation

Duchenne muscular dystrophy (DMD) is an X-linked disorder that results in progressive degeneration and loss of function of striated muscles due to the accumulation of fibrosis in lieu of functional muscle tissue. Approximately one in 5,000 boys are born with DMD, with life expectancy in the 20s without optimal care guidelines to help preserve cardiopulmonary function until the 30s or even early 40s (Duan et al., 2021). The disease results from mutations in the dystrophin gene (*DMD*), which encodes a 427 kDa sarcolemmal structural protein critical for distributing contractile forces by linking the sub-sarcolemmal actin cytoskeleton of cardiomyocytes and myofibers to the extracellular matrix (Ahn and Kunkel, 1993). Dystrophin interacts with the extracellular matrix to help maintain sarcolemmal integrity through direct interactions and localization of the dystrophin-associated glycoprotein complex (DAPC or DGC). The DGC incudes β-dystroglycan, ⍺-dystroglycan, ⍺/β/γ/δ-sarcoglycans, sarcospan, ⍺/β1/β2-syntrophins, ⍺/β-dystrobrevins, and neuronal nitric oxide synthase (nNOS) (Michele and Campbell, 2003). In DMD, when dystrophin and the DGC are not present, striated muscle membranes are susceptible to contraction induced injuries. Ongoing damage of cardiomyocytes and skeletal muscle myofibers results in continuous induction of inflammation, which plays a critical role in cardiac muscle repair and skeletal muscle regeneration (Tidball, 2017; Tidball et al., 2018). However, chronic induction of innate (myeloid) and adaptive (lymphoid) immunological processes can exacerbate pathology by promoting degeneration and impeding healing (Rosenberg et al., 2015).

Cutting edge genetic medicines for DMD are in clinical trials or pre-clinical development including exon-skipping, gene therapy, and genome editing (Fortunato et al., 2021). Gene replacement strategies using adeno-associated virus (AAV) to deliver a packageable form of dystrophin, called micro-dystrophin, are in numerous clinical trials following decades of preclinical studies demonstrating efficacy in skeletal muscles (Gregorevic et al., 2004; Gregorevic et al., 2008; Duan, 2018; Crudele and Chamberlain, 2019; Ramos et al., 2019). AAV-micro-dystrophin also successfully transduces cardiac muscle and improves function in a DMD heart failure mouse model (Gregorevic et al., 2004; Howard et al., 2021a). However, genetic therapies will not restore normal levels of full-length dystrophin, leaving at least residual inflammation that must be targeted to prevent exacerbation of disease pathology (Angelini et al., 2022).

The standard of care for DMD is long-term glucocorticoid receptor (GR) agonist treatment. Activation of GR suppresses inflammatory activities in both innate and adaptive immune cells (Baschant and Tuckermann, 2010). Prednisone, the most common glucocorticoid prescribed to DMD patients, delays ventilator use and modestly increases skeletal muscle strength and ambulation, although the side effects from long term use are numerous (Connolly et al., 2002; Bach et al., 2010; Hussein et al., 2010). Paradoxically, long-term glucocorticoid use causes muscle atrophy in patients without muscle diseases (Schakman et al., 2013). Treating DMD patients intermittently instead of continuously with prednisone helps alleviate side effects, but alternative therapies are needed to improve patient outcomes even with the emergence of genetic medicines (Beenakker et al., 2005; Escolar et al., 2011; Quattrocelli et al., 2017).

## Mineralocorticoid Receptors and Antagonists in Inflammation and DMD Cardiomyopathy

Mineralocorticoid receptors (MR) are ligand-activated transcription factors that regulate gene expression and non-genomic functional activity in numerous cell types in health and pathology (Gomez-Sanchez and Gomez-Sanchez, 2014). MR is part of the nuclear receptor superfamily; therefore, MR resides in the cytosol where upon activation by the endogenous hormonal MR agonists aldosterone or cortisol, the receptors dimerize, translocate to the nucleus, and bind DNA to induce gene expression (Hellal-Levy et al., 2000). The gene encoding human MR, *NR3C2*, contains 10 exons on chromosome 4q31.1. The presence of tissue-specific MR isoforms are thought to be regulated by different promoter sequences (Morrison et al., 1990; Zennaro et al., 1995). MR shares significant sequence homology with GR, progesterone receptor, and androgen receptor. However, the immediate common ancestor is still highly debated (Arriza et al., 1987; Hu and Funder, 2006). MR has an N-terminal domain, DNA-binding domain, and hinge connecting to a C-terminal ligand binding domain (Rogerson et al., 2004). Post-translational modifications (PTMs) on MR further modulate receptor localization, stability, and ligand binding, and include acetylation, phosphorylation, sumoylation, oxidation, and ubiquitylation (Faresse, 2014).

MR’s significant homology with glucocorticoid receptors contributes to the shared binding capacity of MR to both mineralocorticoids and glucocorticoids (Pippal and Fuller, 2008). Aldosterone, the endogenous mineralocorticoid, is secreted by adrenal glands via production in the zona glomerulosa by cytochrome P450 Family 11 Subfamily B Member 2 (CYP11B2), and is essential for regulation of sodium reabsorption and potassium excretion to control blood pressure. Aldosterone production is regulated by the renin-angiotensin-aldosterone system (RAAS) and activates MR in kidney nephrons. Aldosterone has a serum half-life of only 20 minutes (Booth et al., 2002). Cortisol binds more frequently to MR than aldosterone due to its higher relative serum concentrations (Fuller et al., 2012). In certain mammalian tissues, such as kidney, aldosterone binding of MR is greatly enhanced by the presence of microsomal 11β-hydroxysteroid dehydrogenase (11β-HSD2), which converts cortisol to non-binding metabolites, thereby enabling aldosterone to bind MR despite lower intracellular concentrations (Edwards et al., 1988). In addition to transcriptional changes downstream from MR, aldosterone also induces several rapid, non-genomic signaling pathways dependent or independent of aldosterone-mediated MR activation, including calcium mobilization and post-translational kinase modifications (Ruhs et al., 2017). MR coactivator and corepressor peptide interactions are thought to enable multiple activation mechanisms, which depend upon whether aldosterone or cortisol is bound (Hultman et al., 2005).

Mineralocorticoid receptor antagonists (MRAs) have demonstrated safe and effective clinical use over decades to treat hypertension and heart failure in patients with cardiovascular disease, primarily through reductions in blood pressure, cardiac load, and fibrosis (Ferrario and Schiffrin, 2015). Significant evidence from pharmacological treatments and conditional gene knockouts in mice in a variety of disease models, including cardiovascular, kidney, central nervous system, and liver diseases, supports targeting immune cell MR signaling to reduce pathology by dampening harmful inflammation and fibrosis. Knockout of myeloid MR or treatment with MRAs during l-NAME/Angiotensin II, myocardial infarction, or aortic constriction reduces cardiac inflammation and improves healing (Usher et al., 2010; Li et al., 2014; Li et al., 2017; Fraccarollo et al., 2019). Diabetic dysfunction and kidney disease are ameliorated by protection against pro-inflammatory, tissue-destructive inflammatory signaling (Han et al., 2006; Belden et al., 2017; Barrera-Chimal et al., 2018). Knockout of myeloid MR during atherosclerosis prevents exacerbation of pathology by diminishing macrophage cytotoxicity and fibrosis deposition (Shen et al., 2014; Sun et al., 2016; Shen et al., 2017). Macrophage pro-inflammatory responses following ischemic stroke and central nervous system autoimmunity are attenuated with myeloid MR knockout (Frieler et al., 2011; Montes-Cobos et al., 2017). Myeloid MR knockout also inhibits hepatic steatosis and resistance to insulin by beneficially modulating inflammation (Zhang et al., 2017).

Cardiomyopathy leading to heart failure has become a major cause of death in DMD patients, overtaking respiratory failure due to earlier use of nighttime ventilation and prophylactic antibiotics to prevent respiratory infections (D'Amario et al., 2017). Preclinical studies in DMD models support that MRAs reduce cardiomyocyte degeneration, improve cardiac muscle force/strain rate and reduce fibrosis (Rafael-Fortney et al., 2011; Lowe et al., 2016; Lowe et al., 2020). Clinically, both non-specific and specific MRAs, spironolactone and eplerenone respectively, inhibit the decline of left ventricular circumferential strain and ejection fraction in DMD patients to a similar degree (Raman et al., 2015; Raman et al., 2019). Based on these studies, MRAs are now given early in DMD cardiomyopathy progression (Villa et al., 2022). However, of great concern for clinical translation, simultaneous treatment of dystrophic mice with glucocorticoids and MRAs reduces MRA efficacy in both cardiac and skeletal muscle due to competition for MR binding (Janssen et al., 2014).

## Mineralocorticoid Receptor Signaling in Skeletal Muscle Injury and Muscular Dystrophy

In addition to improvements in cardiomyopathy in DMD patients and dystrophic mice, MRAs also improve pathology and function of dystrophic skeletal muscles in mice. Early treatment with MRAs lead to reductions in myofiber degeneration, inflammation and fibrosis, increases in diaphragm specific force, and improved resistance to eccentric contractions in *extensor digitorum longus* muscles (Rafael-Fortney et al., 2011; Lowe et al., 2016; Lowe et al., 2020). Functional improvements cannot be reproduced by treatment with upstream inhibitors of the RAAS system alone (Lowe et al., 2015). Since dystrophic mice, particularly *mdx*, do not recapitulate the severity of human pathology, exercise and aging were employed to exacerbate the phenotype to obtain a more patient-relevant analysis of the benefits of MRAs. However, neither methodology was successful in worsening the phenotype of the *mdx* mouse model (Lowe et al., 2018).

MR is present in normal skeletal muscles, including diaphragm, quadriceps, *tibialis anterior*, gastrocnemius, soleus, and *extensor digitorum longus* (Chadwick et al., 2015). Myoblasts and differentiated myotubes from mice and primary myoblasts and myotubes from humans also produce MR. Levels of MR do not change in aldosterone- or MRA-treated dystrophic mouse skeletal muscle and normal human myotubes (Chadwick et al., 2015; Lowe et al., 2016). MRA treatment changes gene expression of numerous genes in muscle fibers, but also leads to numerous additional changes in inflammatory and fibrotic genes within the dystrophic muscle microenvironment (Chadwick et al., 2015; Chadwick et al., 2017a; Chadwick et al., 2017b).

Protein levels of 11β-HSD2 are present at increased levels in dystrophic muscles, supporting that MR becomes more aldosterone selective in muscular dystrophy (Chadwick et al., 2016). 11β-HSD2 protein is present in primary myotubes from normal humans and normal and *mdx* mice. Gene expression of numerous enzymes required for aldosterone synthesis are also found in dystrophic skeletal muscle and muscle-isolated leukocytes from wild-type mice (Chadwick et al., 2016). The primary enzymes involved in aldosterone synthesis, CYP11B1 and CYP11B2 (aldosterone synthase), are present at the protein level in wild-type skeletal muscle and CYP11B2 is upregulated in dystrophic skeletal muscles. Immunohistochemistry of CYP11B2 demonstrates co-localization with the myeloid cell marker CD11b in dystrophic quadriceps, indicating local immune cell secretion of aldosterone. Aldosterone concentrations are significantly higher in dystrophic quadriceps and diaphragms relative to wild-type, confirming the upregulated CYP11B2 enzyme is actively secreting aldosterone within dystrophic muscle infiltrated with inflammatory cells (Chadwick et al., 2016). These observations help explain MRA efficacy in muscular dystrophy and have implications for understanding their potential use in other diseases where local aldosterone production may occur.

Stabilization of myofiber membranes that likely leads to protection against eccentric contraction-induced injuries is also observed with laser injury assays of myofiber bundles isolated from dystrophic mice treated with MRAs (Chadwick et al., 2017a). Surprisingly, short *ex vivo* MRA treatment also stabilizes membranes in isolated myofiber bundles in laser injury assays (Hauck et al., 2019b). Membrane stabilization MRA effects are independent of myofiber MR, supporting rapid, non-genomic benefits of MRAs via a novel mechanism (Hauck et al., 2019b).

When comparing the transcriptomic effects of non-specific MRAs (spironolactone), specific MRAs (eplerenone), and DMD standard-of-care glucocorticoids (prednisolone) using microarray gene expression analysis of quadriceps muscles derived from treated dystrophic mice, many of the conserved genes between groups are those involved in transcriptional regulation and the immune response (Chadwick et al., 2017a). Similar experiments on normal human myotubes show an overlap in gene expression between aldosterone and prednisolone treatment, indicating glucocorticoids may be activating similar transcriptional profiles to aldosterone via MR signaling (Chadwick et al., 2017b). Both spironolactone and eplerenone lead to similar histological and functional skeletal muscles outcomes and gene expression profiles in dystrophic mice (Lowe et al., 2016; Chadwick et al., 2017a).

Since numerous cell types in the muscle microenvironment express MR, optimization of MRA use is dependent upon understanding how MR signaling contributes to DMD pathology in a cell-specific manner. Myofiber-specific MR knockout on the *mdx* background improves diaphragm specific force and reduces fibrosis (Hauck et al., 2019b). However, since MRAs stabilize membranes independent of MR, myofiber MR knockout does not fully recapitulate MRA treatment.

Defining myofiber MR signaling during acute muscle injury also elucidates mechanistic insights contributing to MRA efficacy in muscular dystrophy. CYP11B2 is transiently expressed during a very brief period at the peak of myeloid cell infiltration after acute muscle injury in normal mice (Hauck et al., 2019a). Conditional knockout of myofiber MR in wild-type mice after muscle injury hampers muscle regeneration by increasing the density of collagen in fibrotic areas as well as reducing myofiber size post-injury, suggesting a delay in wound healing. Increased infiltration of immune cells expressing CYP11B2 is also observed, supporting that MR activation by aldosterone contributes to efficient coordination of regeneration during the skeletal muscle repair process. Treatment with MRAs has similar effects, suggesting that MRAs in chronic versus acute skeletal muscle injuries have contrasting effects (Hauck et al., 2019a).

Myeloid cells are the predominant immune cell found within DMD patient and dystrophic mouse skeletal muscles, and myeloid MR signaling is known to contribute to other disease pathologies as discussed above (Rosenberg et al., 2015; Tidball et al., 2018). Therefore, investigation of MR in innate immune cells may further define the role of MR signaling in the muscle injury microenvironment. Since the diaphragms of dystrophic mice accumulate more fibrosis than other skeletal muscles, we optimized novel skeletal muscle immune cell isolation techniques that enabled statistically powered analysis and comparisons between limb and respiratory muscles in myeloid MR knockout mice on the *mdx* background (Ishizaki et al., 2008; Zhou et al., 2008).

Most previous studies investigating dystrophic skeletal muscle inflammation using flow cytometry relied on density-dependent centrifugation techniques to enrich for immune cells while simultaneously removing debris from skeletal muscle collagenase digestions. However, since the Ficoll gradients used in these preparations, such as Histopaque and Lympholyte, are optimized to capture circulating immune cells exhibiting a narrow range of sizes and granularities, these polysaccharides fail to retrieve a large percentage of the morphologically diverse immune cells that extravasate and proliferate within dystrophic skeletal muscles. Indeed, the pellets typically discarded from these isolations contain numerous immune cell populations (Howard et al., 2021b). Instead of losing yield and biasing results, a back-gating strategy of the pan-leukocyte marker CD45 post-flow cytometry was developed. This method demonstrates that dystrophic diaphragms contain a lower percentage of F4/80^Hi^ transcriptionally active macrophages, but a higher percentage of F4/80^Lo^ macrophages compared to quadriceps muscles from limbs (Howard et al., 2021b). Macrophages expressing high levels of F4/80 upregulate insulin-like growth factor 1 (IGF-1) during mouse skeletal muscle recovery following ischemia-reperfusion injury, and intramuscular injection of F4/80^Hi^ macrophages enhances healing (Hammers et al., 2015). High F4/80 macrophages are also immunosuppressive during regeneration following acute skeletal muscle injury (Arnold et al., 2007). Therefore, the lack of F4/80^Hi^ macrophages during peak diaphragm necrosis may contribute to more advanced fibrosis relative to limb skeletal muscles in commonly used dystrophic mouse models.

Interestingly, myeloid MR knockout in *mdx* and injured wild-type skeletal muscles worsened pathology and prolonged damage, respectively (Howard et al., 2022). Numerous chemokines and cytokines are increased with myeloid MR knockout in *mdx* quadriceps, but are decreased in the diaphragm. Inflammatory signaling is modestly affected in acutely injured myeloid MR knockout muscles, but not to the extent of the myeloid MR knockout on the *mdx* dystrophic background. Changes in myofiber regeneration are not observed in the quadriceps muscles. However, myeloid MR knockout leads to increased diaphragm fibrosis in *mdx* mice. Removing myeloid MR may impact the immune-mediated regeneration response of the muscles to heal correctly in chronically injured dystrophic skeletal muscle. Macrophages displaying C-C motif chemokine receptor 2 (CCR2) are reduced in injured myeloid MR knockout wild-type muscles, which correlates with an increase in ongoing myofiber damage. In addition, CYP11B2 is upregulated in myofibers post-injury, emphasizing a role for aldosterone-mediated MR signaling within the myeloid cells to facilitate muscle repair (Howard et al., 2022).

## Discussion

**FIGURE 1:**
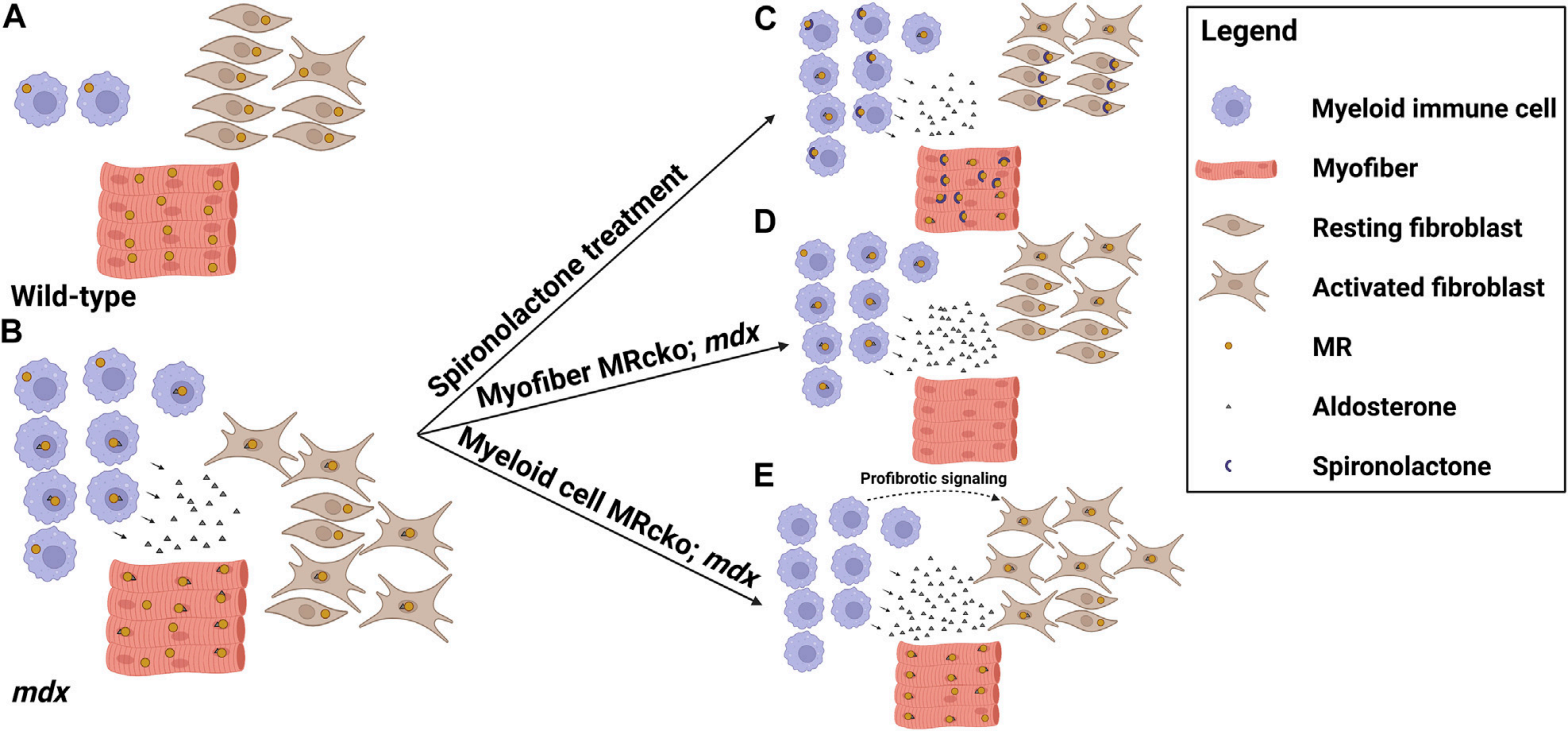
MR signaling in the dystrophic skeletal muscle microenvironment. Model showing the distribution of cell types, mineralocorticoid receptor (MR) expression, and aldosterone production in the striated muscle microenvironments of **(A)** wild-type mice and **(B)** dystrophic *mdx* mice, and the effects on these parameters by altering MR signaling in **(C)** *mdx* mice treated with the MR antagonist spironolactone, **(D)** *mdx* mice with a myofiber-specific MR knockout, and **(E)** *mdx* mice with a myeloid cell-specific MR knockout. Spironolactone-treated dystrophic mice display reduced inflammation, fibrosis, and overall damage in response to MR sequestration and inhibition by spironolactone. Myofiber MR knockout in the increased aldosterone concentration present in the *mdx* muscle microenvironment recapitulates some of the beneficial effects of spironolactone treatment, including reduced fibrosis and increased muscle force. Myeloid cell MR knockout in dystrophic muscle increases fibrosis, possibly due to profibrotic signaling from the MR-deficient myeloid cells contributing to fibroblast activation (Figure was made with Biorender).

MRAs are effective small molecule therapies for the treatment of DMD cardiomyopathy and demonstrate great efficacy on skeletal muscle pathology in preclinical studies. These drugs improve all of the steps involved with the pathogenesis of dystrophic muscles and improve membrane integrity, inflammation and fibrosis, and cellular damage. Cell-specific MR knockouts have helped to define the mechanisms of action of these drugs on dystrophic muscles. Knockout of myofiber MR signaling contributes to muscle force improvements and at least some of the anti-fibrotic effects observed with MRAs (Figure 1). In contrast, knockout of myeloid MR worsens fibrosis in dystrophic muscles (Figure 1). Future studies will be needed to define the specific profibrotic signals involved.

The mechanisms by which MRAs stabilize myofiber membranes through an MR-independent mechanism remain elusive. Recently, inhibition of pannexin 1 channels by the steroidal MRA spironolactone was shown to be a novel mechanism for reducing hypertension. (Good et al., 2018). Whether pannexin 1 represents a potential mechanism for spironolactone’s ability to stabilize dystrophic muscles independently of MR, and beneficial effects from steroidal and non-steroidal MRAs remain to be investigated.

Since glucocorticoids are the standard-of-care for DMD but compete with MRAs for binding MR, alternating their use may prove to be more beneficial for DMD patients than either drug alone. Direct comparisons of the effects of glucocorticoids and MRAs on inflammation will help to identify the optimal use of each of these drugs. Although genetic medicines are promising, adjunct medications will need to be utilized that target inflammation and fibrosis since low-grade muscle inflammation will continue (Kupatt et al., 2021). Further research is required to delineate all cell-specific mechanisms of MR in chronic and acute skeletal muscle injuries. Future work to dissect the role of MR signaling on fibroblasts in chronic and acute muscle injuries will also contribute to optimizing MRA treatment timing for different skeletal muscle pathologies.

Local aldosterone production in skeletal muscle observed from inflammatory myeloid cells and possibly myofibers themselves, as well as previous observations of local aldosterone production in kidney, heart, blood vessels and brain, support the potential use of MRAs for a wider variety of tissue injuries (Kayes-Wandover and White, 2000; MacKenzie et al., 2000; Gomez-Sanchez et al., 2004; Takeda, 2004; Xue and Siragy, 2005). Whether myeloid cell aldosterone production is a more common mechanism of local MR signaling in pathologies involving inflammation remains to be investigated. In addition, MR activation by local production of glucocorticoids also represents an under-investigated area. Future studies of MR signaling may expand the use of MRAs for a larger variety of diseases.
